# Does incentivising pill-taking ‘crowd out’ risk-information processing? Evidence from a web-based experiment^☆^

**DOI:** 10.1016/j.socscimed.2014.01.020

**Published:** 2014-04

**Authors:** Eleni Mantzari, Florian Vogt, Theresa M. Marteau

**Affiliations:** a Health Psychology Section, King's College London, London, UK; b Institute of Pharmaceutical Science, King's College London, London, UK; c Behaviour and Health Research Unit, University of Cambridge, Cambridge, UK

**Keywords:** Financial incentives, Risk-information, Side-effects, Risks, Pill-taking, Health incentives, Medication adherence

## Abstract

The use of financial incentives for changing health-related behaviours raises concerns regarding their potential to undermine the processing of risks associated with incentivised behaviours. Uncertainty remains about the validity of such concerns. This web-based experiment assessed the impact of financial incentives on i) willingness to take a pill with side-effects; ii) the time spent viewing risk-information and iii) risk-information processing, assessed by perceived-risk of taking the pill and knowledge of its side-effects. It further assesses whether effects are moderated by limiting cognitive capacity. Two-hundred and seventy-five UK-based university staff and students were recruited online under the pretext of being screened for a fictitious drug-trial. Participants were randomised to the offer of different compensation levels for taking a fictitious pill (£0; £25; £1000) and the presence or absence of a cognitive load task (presentation of five digits for later recall). Willingness to take the pill increased with the offer of £1000 (84% vs. 67%; OR 3.66, CI 95% 1.27–10.6), but not with the offer of £25 (79% vs. 67%; OR 1.68, CI 95% 0.71–4.01). Risk-information processing was unaffected by the offer of incentives. The time spent viewing the risk-information was affected by the offer of incentives, an effect moderated by cognitive load: Without load, time increased with the value of incentives (£1000: *M* = 304.4sec vs. £0: *M* = 37.8sec, *p* < 0.001; £25: *M* = 66.6sec vs. £0: *M* = 37.8sec, *p* < 0.001). Under load, time decreased with the offer of incentives (£1000: *M* = 48.9sec vs. £0: *M* = 132.7sec, *p* < 0.001; £25: *M* = 60.9sec vs. £0: *M* = 132.7sec, *p* < 0.001), but did not differ between the two incentivised groups (*p* = 1.00). This study finds no evidence to suggest incentives “crowd out” risk-information processing. On the contrary, incentives appear to signal risk, an effect, however, which disappears under cognitive load. Although these findings require replication, they highlight the need to maximise cognitive capacity when presenting information about incentivised health-related behaviours.

## Highlights

• Concerns exist that health incentives undermine risk-information processing.
• We assessed these concerns in the context of taking a pill with side-effects.
• Financial incentives did not undermine the processing of risk-information.
• Incentives increased risk-information viewing time, implying a cautionary effect.
• This cautionary effect disappeared under cognitive load.

## Introduction

Financial incentives are increasingly being considered and used in health policies in the UK and elsewhere, in an attempt to improve health-related behaviours (Le Grand, 2008; Marteau, Ashcroft, & Oliver, 2009). They have been used most often in low and middle income countries as part of programmes which aim to reduce poverty and health inequalities. These programmes use conditional cash transfers that are delivered to families, if certain health and educational behaviours have been performed (Lagarde, Haines, & Palmer, 2007). They have also been used in high-income countries to target some health behaviours, including tobacco use, unhealthy eating and lack of physical activity (e.g. APM Health Europe, 2007; North East Essex NHS Trust, 2009), as well as poor management of chronic conditions (e.g. Claassen, Fakhoury, Ford, & Priebe, 2007; World Bank, 2008). Most financial incentive schemes involve the offer of a reward, such as a cash payment, a voucher or a prize, which is delivered if a pre-specified behaviour or outcome has been achieved. Other schemes involve the use of a ‘deposit-contract’ whereby individuals pledge their own money, which they lose if they fail to meet their goals.

Unlike most interventions designed to change health behaviours, the use of financial incentives raises particular concerns regarding their potentially adverse effects on the quality of people's decisions to engage in incentivised behaviours. This is particularly relevant to behaviours associated with adverse side-effects, such as taking certain medicines, receiving immunisation, and attending screening appointments. The specific concern is that the prospect of receiving a financial reward could result in the risks associated with an incentivised health behaviour being overlooked (Marteau et al., 2009). There are two possible ways this could occur: first, financial incentives might lead people to ignore or not process risk-information; second, people offered financial incentives might process risk- information but might not feel that it applies to them, and therefore continue to perceive the risks to themselves as low compared to those not offered incentives. Results from a recent randomised controlled trial, in which teenage girls were offered shopping vouchers worth £45 (€56; $73) for undergoing three doses of the HPV vaccination, did not find the offer of financial incentives to undermine the quality of people's decisions to engage in an incentivised health-related behaviour (Mantzari, Vogt, & Marteau, in press). These conclusions were based on an assessment of girls' ability to make informed choices, as measured by their attitudes towards the HPV vaccination and their knowledge of the HPV vaccination's consequences on health. Knowledge of the vaccination's adverse side-effects was not assessed. Consequently, findings do not allow inferences to be made about whether or not the offer of a financial reward results in the risks associated with the incentivised behaviour being overlooked (Marteau et al., 2009).

We are unaware of any studies that have assessed the impact of financial incentives on the processing of risk-information associated with an incentivised health-related behaviour. Research within two conceptually analogous domains could help elucidate the uncertainty. The first involves the use of payments for live organ donations, which have been criticised for undermining donors' ability to calculate the related risks (e.g. Becker & Elias, 2007; Olbrisch, Benedict, Haller, & Levenson, 2001). Partial support for this claim derives from studies investigating the economic and health consequences of selling kidneys in India (Goyal, Mehta, Schneiderman, & Sehgal, 2002) and Pakistan (Naqvi, Ali, Mazhar, Zafar, & Rizvi, 2007). Findings show that the majority of vendors were very poor and sold their organs to pay off debts, but would not recommend others to do the same. This could be taken as an indication of regret and interpreted as sellers having been unaware of the negative consequences associated with organ donation. It is not clear, however, whether that was because they were inadequately informed of the likely outcomes or because the prospect of money led them to ignore the risks or perceive them as not being applicable to them. Recent research shows that as the risk of renal failure increases, individuals become less willing to donate kidneys, regardless of the level of payment offered, therefore suggesting that financial incentives do not blind people to the risks of living kidney donation (Halpern et al., 2010).

The second related research area involves the use of financial incentives for participation in research, including clinical trials. Payments increase individuals' willingness to participate in research (Bentley & Thacker, 2004; Singer, Groves, & Corning, 1999; Slomka, McCurdy, Ratliff, Timpson, & Williams, 2007). They have, however, been criticised for being undue inducements (Dickert & Grady, 1999) that alter decision-making processes, such that the side-effects of participating are not fully considered (Dickert, Emanuel, & Grady, 2002), and risks are overlooked (Grant & Sugarman, 2004; London, 2005), thus leading individuals to expose themselves unwittingly to the possibility of harm (McNeill, 1997). These concerns are largely hypothetical with the evidence about how participation payments influence perceived risk and decision-making processes being scarce. The few studies that have been conducted in the area suggest that compensation does not lead people to neglect research risks (Bentley & Thacker, 2004; Dunn, Kim, Fellows, & Palmer, 2009; Halpern, Karlawish, Casarett, Berlin, & Asch, 2004; Singer & Couper, 2008). Specifically, it has been found that people make rational trade-offs between risk and benefit. Although they are willing to accept more risk in return for more money, this does not blind them to risk or distort their judgments (Bentley & Thacker, 2004; Dunn et al., 2009; Halpern et al., 2004; Singer & Couper, 2008). On the contrary, participation payments could signal risk and increase vigilance and information seeking when the amount offered is high. In one study, participants were allocated to view information regarding either a trial that involved drawing blood or a trial that involved Transcranial Magnetic Stimulation and were offered either $25, $100 or $1000 for participation. Findings showed that compared to the low-payment scenarios, the offer of a high payment (i.e. $1000) increased participants' willingness to participate, but also increased perceived risk and the time they spent viewing the risk-information (Cryder, London, Volpp, & Loewenstein, 2010).

Although the above findings highlight some of the potential effects of financial incentives on the processing of risk-information, certain limitations associated with the design of the studies do not allow firm conclusions to be drawn. These include first, a failure to incorporate conditions of no payment, which prevents an assessment of the absolute effect of financial incentives on risk-information processing; second a lack of measures of individuals' knowledge of risks. It has been suggested that when motivated by cash payments, individuals may have less interest in assessing or comprehending study details, reading consent forms or attempting to understand the research aims and related risks (Grady, 2005). Accordingly, an assessment of the impact of financial incentives on individuals' knowledge of risks is essential. A third limitation of existing studies stems from the reliance on hypothetical scenarios, of which participants were aware. Only one study (Cryder et al., 2010) led individuals to believe that they were responding to information of an actual trial, in which they could participate.

In addition to the above, to our knowledge no studies have assessed the potential role of limited cognitive capacity on the impact of financial incentives on risk-information processing. In real-life situations, the cognitive resources of some people invited to decide about engaging in incentivised behaviours are often overloaded with matters of daily living. Cognitive capacity can also be affected by the way in which information (e.g. the design and format) about the potential adverse consequences of incentivised health-related behaviours is given, which could overload working memory, thus inducing cognitive load and reducing the amount of cognitive resources available for processing the informational content (Chandler & Sweller, 1991; Sweller, 1994; Sweller, Van Merrienboer, & Paas, 1998). Consistent with the assumptions of “dual-processing” models of decision-making (e.g. Strack & Deutsch, 2004), findings demonstrate that cognitive load inhibits activation of the reflective system that generates behavioural decisions based on reasoning, judgment and knowledge about facts and values and increases activation of the impulsive system that elicits behaviour through associative links (Hinson, Jameson, & Whitney, 2002; Shiv & Fedorikhin, 1999). Consequently, under cognitive load, people have less ability to process risks and rely on heuristics to make satisfactory decisions with minimal effort (Friese, Hofmann, & Wänke, 2009; Hofmann, Gschwendner, Friese, Wiers, & Schmitt, 2008; Whitney, Rinehart, & Hinson, 2008). From this it seems possible that the potential adverse effects of financial incentives on risk-information processing are amplified under conditions of cognitive load.

In sum, given the scarcity of empirical studies in the area, in addition to the above shortcomings, further research is needed, particularly experiments, to illuminate the mechanisms by which financial incentives influence people's decision-making mechanisms, including the processing of risk-information.

### The present study

The aim of this study was to assess the impact of financial incentives on the quality of decisions to consume medicine with potential side-effects. This specific context was chosen because financial incentives have been used to improve medication compliance (e.g. DeFulio et al., 2012; Hill & Ramachandran, 1992; Morisky et al., 2001; Pilote et al., 1996; Sorensen et al., 2007; Volpp et al., 2008), yet their impact on the processing of side-effects associated with most medicines has thus far remained unstudied.

The study specifically evaluates the impact of low-value (£25) and high-value financial incentives (£1000) on i) willingness to consume a pill with side-effects, ii) the time spent viewing the pill-related information, and iii) risk-information processing, as assessed by a) the level of perceived risk associated with consuming the pill and b) knowledge of its side-effects. These specific incentive values were chosen to model the low and high value incentives used by Cryder et al. (2010) (i.e. $25 and $1000), which is the only other study we are aware of which led individuals to believe that they were responding to information of an actual trial, in which they could participate. The study further assesses the extent to which any effects are moderated by cognitive load.

### Hypotheses

1) The offer of financial incentives increases the proportion of individuals who are willing to take the pill.
2) The offer of a low-value financial incentive does not increase the time spent viewing the pill-related information compared to no incentive.
3) The offer of a high-value financial incentive increases the time spent viewing the pill-related information compared to no incentive.
4) The offer of a low-value incentive does not affect risk-information processing.
5) The offer of a high-value incentive affects risk-information processing.
6) The impacts of financial incentives on the time-spent viewing risk information and risk-information processing are amplified under conditions of cognitive load.

## Methods

### Design and overview

The present study is a web-based experiment. Participants were recruited online under the pretext of being screened for a fictitious trial examining the impact of a new cognitive-enhancing pill on memory. Using a 3 × 2 factorial design, participants were randomised to view webpages including the offer of different levels of financial incentives for taking the fictitious pill (no incentive; £25; £1000) and the presence or absence of a task intended to induce cognitive load.

### Participants

Participants comprised two-hundred and seventy-five (*n* = 275) staff, students and alumni of universities based in London, UK. Table 1 presents participants' demographic characteristics. Their mean age was 25.3 years, ranging from 18 to 56 years and 52% were female. The majority was White (59%), with 29% classified as Asian and 5% as Black. Most participants were students (75%), working towards a first or second degree, 17.5% were in full-time or part-time employment and 6% were unemployed or homemakers.

### Recruitment and randomisation

Participants were recruited online through circular emails sent to the contacts of a large participant database held by the Behavioural Research Lab at the London School of Economics, and to the staff and students of two schools (School of Law and School of Arts and Humanities) of King's College London, UK. The emails informed participants of the existence of a fictitious trial assessing the impact of a new cognitive-enhancing pill on memory. Interested individuals were invited to complete the fictitious trial's eligibility screening process, by clicking on a link contained within the email, which led to the study website. They were informed that they would receive a shopping voucher worth £10 for completing the screening process. Upon clicking the link, participants were randomised to view one of six webpages, differing in the level of incentives offered for consuming the fictitious pill (reimbursement of travel expenses; reimbursement of travel expenses plus £25; or reimbursement of travel expenses plus £1000) and the inclusion or exclusion of a task intended to induce cognitive load. The task consisted of the presentation of a five-digit number, which participants had to remember and recall at a later stage during the study. The randomisation resulted in six study groups.

### Measures

#### Willingness to take the pill

Willingness to take the fictitious pill was assessed by requesting participants to specify whether they wished to participate in the fictitious trial. They chose one of four available options: a) *yes definitely*; b) *yes, probably, but I would like to discuss further with a member of the research team*; c) *no, probably not*, *but I would like to discuss further with a member of the research team;* d) *no, definitely not*. Because consumption of the pill was described as the central component of the fictitious trial, willingness to participate was considered equivalent to affirmative disposition to taking the pill.

#### Time spent viewing risk-information

The time participants remained on the webpage presenting the pill-related information was recorded in milliseconds.

#### Risk-information processing

##### Perceived risk

Perceived risk associated with taking the fictitious pill was measured using a questionnaire consisting of:

i) two items assessing the *perceived likelihood* of experiencing any or all of the pill's side-effects, rated on a five-point scale ranging from 1: extremely unlikely to 5: extremely likely.
ii) two items assessing the *perceived severity* of experiencing any or all of the pill's side-effects, rated on a five-point scale ranging from 1: extremely good to 5: extremely bad.

Average perceived likelihood and perceived severity scores were multiplied to obtain perceived risk scores.

##### Knowledge of side-effects

Knowledge of the pill's side effects was measures in three ways:

i) participants were requested to freely recall as many of the pill's side effects as they could and to indicate wherever possible, whether these were described in the pill-related information as “very common”, “common” or “uncommon”. One point was scored for every correctly listed side-effect. Another point was given if the side-effect was accompanied by a correct description of its frequency. Scores ranged from 0 to 24.
ii) participants were requested to choose from a list of possible side-effects the ones that were described in the related information as requiring medical attention. The possible options were: a) skin rash; b) fast heartbeat; c) speech problems; d) psychiatric reactions; and e) memory loss. The correct answers were (a) and (d) and participants were scored one point for correctly choosing each. Selection of all options was taken as an indication of participants' guessing and resulted in one point being deducted. Scores ranged from 0 to 2.
iii) participants were presented with six situations for which they were required to specify whether the pill should be taken with caution or should not be taken at all. The situations were: a) pregnant and/or breastfeeding women (should not be taken); b) people with high blood pressure (should not be taken); c) people with liver problems (should be taken with caution); d) people with irregular heartbeats (arrhythmias) (should not be taken); e) people with a history of mental health problems (should be taken with caution); f) people with a history of substance abuse (should be taken with caution); Participants were scored one point for each correct specification. Scores ranged from 0 to 6.

To obtain an aggregated measure of knowledge, scores from the three measures were added together. Aggregated scores ranged from 0 to 32. (Cronbach's alpha = 0.44).

### Procedure

Upon entering the study website, all individuals were presented with information about the fictitious trial, including its aim, which they were told was to assess the immediate impact of a new analeptic drug called Modagil, on non-sleep deprived individuals' performance on memory-related tasks. Participants were informed that the fictitious screening process would involve completion of a series of questionnaires and would result in receipt of a £10 Amazon voucher. They were further informed that completion of the trial would involve visiting a lab, taking a single dose (one pill) of the drug and completing a number of simple memory-related tasks. Participants also read information regarding their compensation for taking part in the trial, which differed according to the group to which they had been allocated (travel expenses; travel expenses and £25; travel expenses and £1000). The subsequent webpage presented participants with information about the fictitious drug Modagil, including its approved and off-label uses, its side-effects, including information about those which require medical attention, and its counter-indications. Although Modagil is not a real drug, most of the information used in the study was modelled on the actual drug Modafinil. A fictitious name was chosen to prevent participants who could not recall the pill's side-effects from finding relevant information on the internet, in order to complete the measures of knowledge. It was confirmed that searching the internet for the term “Modagil” would only yield information regarding the trademarking registration for this brand by a pharmaceutical company. This was considered appropriate, as it would potentially reinforce participants' belief in the credibility of the fictitious drug. Informed consent was provided by all participants via the next webpage. Participants allocated to the groups exposed to the cognitive load task subsequently viewed a webpage presenting them with five randomly selected digits, which they were instructed to memorise for later recollection. This “cognitive load” manipulation has been used previously (Hinson, Jameson, & Whitney, 2003; Shiv & Fedorikhin, 1999; Whitney et al., 2008). In the next webpage participants were requested to complete a questionnaire assessing demographic variables such as age, gender, level of education and occupation. Subsequently, they were required to complete the measures of perceived risk and knowledge of the pill's side effects and were asked to specify their willingness to take part in the fictitious trial. To prevent participants from re-visiting the pill-related information in order to complete the measures of knowledge, the ‘back’ function of the website was disabled. Upon completion of all the measures, participants were debriefed and informed of the true aims of the study. The study was approved by the London School of Economics Research Ethics Committee, Reference Number 203/26.06.2012.

### Statistical analysis

To increase power, responses regarding participants' willingness to take the pill were collapsed to create two outcomes: Yes and No. Time and knowledge scores were log-transformed to correct for their non-normal distribution. To assess the effects of financial incentives and cognitive load on willingness to take the pill, logistic regression analysis was conducted. To assess the effects of financial incentives and the moderating role of cognitive load on the time spent viewing the pill-related information, perceived risk and knowledge of the pill's side-effects, univariate analyses of variance were conducted for each outcome variable separately. Due to the low Cronbach's alpha between items assessing knowledge, the effects of the independent variables on each component of the knowledge scale was also assessed. Where analyses revealed significant interactions between the two independent variables, these were explored using simple main effects analyses and pairwise comparisons. Obtained *p*-values were adjusted for the number of comparisons using the Bonferroni correction. All tests were assessed at the 5% level of significance.

### Power calculations

Given the £10 expense associated with each participant, the total sample size of 275 individuals was determined by the availability of resources. Post hoc analyses using GPower 3.1 revealed that the study had 80% power to detect a small effect (*d* = 0.18) at the 5% significance level between groups on outcomes subjected to univariate analysis of variance, i.e. those relating to the time-spent viewing risk-related information, perceived risk, and knowledge. Based on the findings of Cryder et al. (2010) relating to an average proportion of 17% of participants offered £25 being willing to participate in the trial (the study did not include a ‘no incentive’ control group), the present study also had 80% power to detect a minimum difference in willingness to take the pill of 18% between groups at the 5% statistical significance level using a two-tailed *χ*^2^ test.

## Results

All groups were comparable in demographic characteristics (age, gender, education, occupation, ethnicity) (Tables 1 and 2).

### Willingness to take the pill

The offer of £1000 increased the proportion of participants who were willing to take the pill (84% vs. 67%; OR 3.66, CI 95% 1.27–10.6, *p* = 0.02). The offer of £25 did not significantly increase the proportion of individuals who were willing to take the pill (OR 1.68, CI 95% 0.71–4.01, *p* = 0.24). Cognitive load did not affect participants' willingness to take the pill (OR 1.24, CI 95% 0.50–3.05, *p* = 0.65) nor did it moderate the impact of financial incentives on willingness to take the pill (Interaction between offer of £1000 and cognitive load: OR 0.46, CI 95% 0.11–1.95, *p* = 0.29; Interaction between offer of £25 and cognitive load: OR 1.25, CI 95% 0.32–4.85, *p* = 0.75).

### Time spent viewing risk-information

The offer of financial incentives had a significant impact on the time spent viewing risk-information, *F* (2, 270) = 7.14, *p* < 0.001. This effect was moderated by cognitive load, *F*(2, 270) = 35.4, *p* < 0.001. The effect of incentive level was significant both in the absence of cognitive load, *F*(2, 270) = 33.1, *p* < 0.001, and in its presence, *F* (2,270) = 9.18, *p* < 0.001. Under no load, participants offered £1000 for taking the pill spent longer time viewing the pill information (*M* = 304.4 s) compared both to those not offered incentives (*M* = 37.8sec), *p* < 0.001, and those offered £25 (*M* = 66.6sec) *p* < 0.001. Those offered £25 also spent more time viewing the information compared to those not offered incentives, *p* < 0.001. Under load, those offered both £1000 (*M* = 48.9sec) and £25 (*M* = 60.9sec) spent less time viewing the information compared to those not offered incentives (*M* = 132.7sec), *p* < 0.001 (for both comparisons). There was no significant difference between the two incentivised groups, *p* = 1.00. Among those not offered incentives, time increased in the presence of load (*M* = 132.7sec) compared to its absence (*M* = 37.5sec), *p* < 0.001. The opposite pattern was observed for those offered £1000 (no load: *M* = 304.4sec; load: 48.9), *p* < 0.001. For those offered £25, there was no difference in time in the absence (*M* = 66.6sec) and presence of cognitive load (load: *M* = 60.9sec), *p* = 0.13.

### Risk information processing

#### Perceived risk

The perceived risk associated with taking the pill was unaffected by the offer of financial incentives, *F* (2, 275) = 2.61, *p* = 0.70. It was also unaffected by cognitive load, *F* (1, 275) = 1.71, *p* = 0.19. Cognitive load did not moderate the impact of financial incentives on perceived risk, *F* (2, 275) = 0.2.08, *p* = 0.13.

#### Knowledge of the side-effects

Knowledge of the pill's side-effects was unaffected by the offer of financial incentives, *F* (2, 259) = 0.15, *p* = 0.86. It was also unaffected by cognitive load, *F* (1, 259) = 0.02, *p* = 0.52. Cognitive load did not moderate time impact of financial incentives on knowledge of the pill's side-effects, *F* (2,259) = 0.12, *p* = 0.47. Each component of knowledge was also unaffected by the offer of financial incentives (Free recall: *F* (2, 252) = 1.36, *p* = 0.26; Knowledge of side-effects requiring medical attention: *F* (2, 250) = 1.96, *p* = 0.14; Knowledge of counter-indications: *F* (2, 265) = 1.65, *p* = 0.19) and cognitive load (Free recall: *F* (1, 252) = 1.59, *p* = 0.21; Knowledge of side-effects requiring medical attention: (*F* (2, 250) = 0.77, *p* = 0.38; Knowledge of counter-indications: (*F* (2, 265) = 0.15, *p* = 0.70), as well as the interaction between financial incentives and cognitive load (Free recall: *F* (2, 252) = 2.15, *p* = 0.12; Knowledge of side-effects requiring medical attention: (*F* (2, 250) = 1.04, *p* = 0.36; Knowledge of counter-indications: (*F* (2, 265) = 0.92, *p* = 0.40).

## Discussion

The offer of £1000 increased the proportion of participants who were willing to take the pill, but the offer of £25 did not. The offer of an incentive did not “crowd out” the processing of risk-information: levels of perceived risk associated with taking the pill and knowledge of its side-effects did not differ between groups. Cognitive load did not moderate the impact of financial incentives on willingness to take the pill or processing of risk-information. The time spent viewing the pill-related information was affected by the offer of financial incentives, an effect moderated by cognitive load: In the absence of load, time increased with the value of incentives, with those offered £1000 spending the longest time viewing the information. Under load, the offer of financial incentives reduced viewing time, with both those offered £25 and £1000 spending less time viewing the information compared to those not offered incentives.

The positive impact of offering £1000 on willingness to take the pill is consistent with predictions and previous research showing that higher payments increase willingness to participate in clinical trials compared to lower payments (Bentley & Thacker, 2004; Cryder et al., 2010; Halpern et al., 2004). Although the offer of £25 increased the proportion of individuals willing to take the pill by 12% (95%CI = −2%–25%) compared to those not offered incentives, this difference was not found to be significant. This finding is contrary to predictions, as well as research showing that incentives as low as £3 ($5) can increase medication compliance (e.g. Bock, Sales, Rogers, & DeVoe, 2001; Chernew et al., 2008; Volpp et al., 2008). This result appears most likely due to a lack of statistical power to detect such an effect. Indeed, post-hoc analyses revealed that the study was powered to detect a minimum difference in willingness to take the pill of 18%. The difference, however, between those offered £25 and those not offered incentives was smaller.

Although the offer of £1000 increased participants' willingness to take the pill, it did not do so by leading them to overlook the related risks. Contrary to existing concerns (Dickert et al., 2002; Dickert & Grady, 1999; Grady, 2005; Grant & Sugarman, 2004; London, 2005; Marteau et al., 2009), financial incentives in this study did not undermine risk-information processing. This is consistent with previous research showing that in hypothetical situations, although people are willing to accept more risk in return for more money, this does not blind them to risk or distort their judgments (Bentley & Thacker, 2004; Dunn et al., 2009; Halpern et al., 2004, 2010; Singer & Couper, 2008). On the contrary, large offers of compensation have been suggested to signal risk (Cryder et al., 2010; Frey & Oberholzer-Gee, 1997) and have been shown by Cryder et al. (2010) to increase information-seeking, as well as perceived risk. Similarly, in the absence of cognitive load those offered large incentives in the present study spent the longest time viewing the risk-information. Most importantly, results from this study also show that even small incentives can signal risk, as indicated by an increase in the time spent viewing risk-information in the absence of cognitive load by those offered £25 compared to those not offered incentives. Contrary to the findings of Cryder et al. (2010), however, no effect was observed on perceived risk in the current study. This apparently conflicting result might reflect the use of different measures of perceived risk by the two studies (i.e. judgements about a medical procedure's riskiness in comparison to other risky activities vs. personal perceived risk associated with consuming the pill). Alternatively, it might represent the different levels of riskiness associated with the procedures in which participants were requested to engage in each of the studies (Transcranial Magnetic Stimulation vs. taking a pill with relatively mild side-effects). This would imply that individuals do not simply equate large incentives with riskiness in an automatic way, but might perceive them as an indication of the need to pay more attention to risk-information. Whether or not this increased vigilance leads to increased levels of perceived risk may depend on the actual riskiness of the related procedures.

In the absence of cognitive load, the offer of large incentives resulted in the greatest increase in the time spent viewing the risk-information, but contrary to predictions, this was not accompanied by an increase in knowledge of the pill's side effects. This raises the question of what people were doing during the additional time they viewed the pill information. There are two possible answers. First, based on the assumption that large payments signal more risk, it is possible that those in the high-incentive group used the additional time to examine the information more carefully for anything that could be alarming, but did not pay additional attention to information they did not perceive as risky. According to this possibility, large incentives would increase knowledge of severe side-effects, rather than of all side-effects in general. As the variation of side-effect severity in the present study was not particularly large, this assumption cannot be tested. Alternatively, it is possible that the lack of differences in knowledge was the result of a ceiling effect. Perhaps the chosen side-effects were particularly easy to remember. Indeed some, such as headache and dizziness, are commonly associated with many medications. Consistent with this assumption, knowledge levels in this study were fairly high in all groups, regardless of the difficulty of the free-recall measure used. Consequently, perhaps the additional time participants spent on the pill-information failed to increase knowledge because they had reached the limits of their working memory capacity.

Consistent with the aforementioned notion that incentives are not equated with risk in an automatic way is the finding that under cognitive load the signalling effect of incentives –implied by increased time spent viewing risk-information– disappeared. In fact, in the presence of cognitive load, those offered £1000 spent the least amount of time viewing the pill-related information. It appears that when cognitive resources are limited, the offer of incentives, especially large ones, can undermine risk-information seeking, perhaps due to the activation of more automatic processes, which narrow individuals focus; more automatic processing has been linked to limited cognitive resources (Hinson et al., 2002; Shiv & Fedorikhin, 1999). However, contrary to previous suggestions that people have less ability to process risks under cognitive load (Friese et al., 2009; Hofmann et al., 2008; Whitney et al., 2008) in the present study, the decreased time spent viewing risk-information under conditions of cognitive load by those offered incentives did not undermine risk-information processing.

The findings from the present study add to the evidence which challenges existing concerns regarding the adverse impact of financial incentives on the processing of risk-information associated with incentivised behaviours. In fact, results suggest that incentives, especially large ones, signal the need for increased attention towards risk-information. Results further suggest that under conditions of cognitive load this signalling effect disappears. As cognitive load is affected by the design and format of information and instructions (Chandler & Sweller, 1991; Sweller, 1994; Sweller et al., 1998), the findings highlight the importance of presenting information about incentivised health-related behaviours and their consequences in a way that maximises cognitive capacity, in order to preserve the signalling effect of incentives and avoid a possible narrowing of attention.

Certain limitations associated with the current study, however, compromise the validity and generalisability of the conclusions that can be drawn. In addition to the aforementioned issues relating to the use of potentially easy-to-remember side-effects and the limited variation in side-effect severity, the present study has other methodological limitations. The first relates to the possible discrepancy between willingness to take the pill and actual behaviour. Although the majority of participants were willing to take the pill, it is not known how many would actually do so. Second, although measures were taken to ensure that the trial appeared credible, it is possible that some participants may have realised its fictitious nature, which could have influenced their responses. As perceived credibility of the trial was not measured, the extent to which this occurred is unknown. The third limitation relates to the generalisabilty of the results. The majority of participants consisted of students registered with research databases who were potentially accustomed to taking part in experiments and may therefore have been more suspicious than the general population. We do not know if similar findings will be obtained with a patient population offered payments to take a medicine. Furthermore, cognitive load information consisted of a 5-digit number rather than information relevant to the medication. It should also be highlighted that the sample size of the study was determined by available resources rather than power calculations. As such, the analysis of the full 3 × 2 factorial design was possibly underpowered in that group sizes were generally under *n* = 50.

In conclusion, the findings from the present study provide no evidence to support the concerns regarding the adverse effects of financial incentives on risk-information processing and the quality of decisions to engage in incentivised behaviours. Low value incentives do not “crowd out” risk-information processing or affect willingness to perform incentivised behaviours. Although large-value incentives increase willingness to engage in incentivised behaviours, their offer signals more risk. This signalling effect disappears when the cognitive capacity to process information is reduced, highlighting the need to maximise cognitive capacity when presenting information about an incentivised health-related behaviour. Despite the many strengths of the study, these findings require replication in future research that will overcome its limitations.

## Figures and Tables

**Table 1:** Demographic characteristics of study participants.

Characteristic	No incentives (*n* = 86)		£25 (*n* = 99)		£1000 (*n* = 90)		Total (*n* = 275)
	No load (*n* = 43)	Load (*n* = 43)	No load (*n* = 41)	Load (*n* = 58)	No load (*n* = 43)	Load (*n* = 47)	
Mean age yrs (sd)	24.4 (8.07)	23.9 (5.16)	25.7 (7.32)	23.9 (7.00)	26.7 (9.74)	25.2 (8.19)	25.0 (7.43)
*Gender*							
Male	44%	49%	41%	44%	51%	55%	48%
Female	56%	51%	59%	55%	49%	45%	52%
*Ethnicity*							
White	49%	63%	58%	52%	67%	66%	59%
Mixed	9%	2%	5%	7%	0%	4%	5%
Asian	37%	28%	27%	36%	21%	23%	29%
Black	2%	5%	10%	2%	5%	6%	5%
*Occupation*							
Student	74%	74%	68%	79%	79%	74%	75%
Employed full-time	14%	16%	19%	7%	16%	15%	15%
Employed part-time	0%	5%	2%	5%	2%	4%	3%
Unemployed	7%	2%	5%	3%	0%	0%	6%
*Education*							
A-levels	14%	12%	15%	19%	14%	17%	15%
Working towards degree	30%	28%	24%	28%	32%	21%	27%
Completed degree	21%	12%	17%	14%	9%	13%	14%
Working towards postgrad	21%	32%	24%	24%	20%	30%	25%
Completed postgrad	14%	14%	17%	12%	23%	17%	16%
*Relationship*							
Single	37%	60%	49%	64%	44%	59%	52%
In relationship	37%	26%	32%	21%	32%	28%	29%
Married	16%	2%	12%	7%	14%	9%	10%

**Table 2:** Means (SD) or % (*n*) of outcome variables for each group.

	No incentives			£25			£1000		
	No load (*n* = 43)	Load (*n* = 43)	Overall (*n* = 86)	No load (*n* = 41)	Load (*n* = 58)	Overall (*n* = 99)	No load (*n* = 43)	Load (*n* = 47)	Overall (*n* = 90)
% Willingness to participate (*n*)	70% (30)	65% (28)	67% (58)	83% (34)	76% (44)	79% (78)	80% (35)	87% (41)	84% (76)
Time (sec) (sd)	37.77 (29.97)	132.7 (137.1)	85.24 (109.6)	66.59 (39.57)	60.93 (74.82)	63.27 (62.48)	304.4 (516.4)	48.94 (28.24)	172.5 (379.8)
Perceived risk (sd)	9.95 (4.15)	9.46 (4.92)	9.70 (4.53)	8.10 (3.26)	8.58 (3.77)	8.38 (3.56)	9.91 (4.45)	7.99 (3.43)	8.90 (4.04)
Knowledge (sd)	12.9 (5.90)	12.5 (5.90)	12.7 (5.87)	13.5 (6.37)	12.1 (5.30)	12.7 (5.79)	12.4 (7.04)	11.7 (4.90)	12.0 (5.73)
